# Ectopic Sebaceous Glands in the Esophagus: A Case Report and Review of Literature

**DOI:** 10.4103/1319-3767.39624

**Published:** 2008-04

**Authors:** Ramachandra V. Bhat, Rupnarayan R. Ramaswamy, Lakshmana Kumar C. Yelagondahally

**Affiliations:** Department of Pathology, Sri R.L. Jalappa Hospital and Research Centre, Tamaka, Kolar-563 101, Karnataka, India; 1 Department of Medicine, Sri R.L. Jalappa Hospital and Research Centre, Tamaka, Kolar-563 101, Karnataka, India

**Keywords:** Esophagus, sebaceous glands

## Abstract

Ectopic sebaceous glands occur rarely in the esophagus. A 65-year-old man presented with a history of discomfort during swallowing since the last 4 months. On upper gastrointestinal endoscopy, multiple wart-like grayish-yellow projections were detected and two of them were biopsied. Microscopically, they proved to be sebaceous glands in the esophagus. Histogenesis of this rare lesion is discussed in this case report.

Sebaceous glands are derived from the ectoderm. Ectopic sebaceous glands have been reported in various sites such as lips and mouth (in Fordyce's disease) eyes, orbits, palms and soles, salivary glands, tongue and larynx. In esophagus, which is an endodermally derived organ, the presence of ectopic sebaceous glands is rare.[1] This condition was described in only 19 living individuals in previous studies.[2–4]

## CASE REPORT

A 65-year-old man presented with a history of discomfort during swallowing since the last 4 months. There was no history of vomiting or hematemesis and weight loss. The patient was not a chronic smoker or an alcoholic. No history of chronic tobacco chewing was reported. General physical examination was unremarkable. Respiratory and cardiovascular systems were normal. Clinically, carcinoma of esophagus was suspected, and upper gastrointestinal (GI) endoscopy was performed.

### Endoscopy findings

On upper GI video endoscopy, multiple (nearly 20) grayish-yellow nodular projections of 2–4 mm diameter were noticed in the middle and lower esophagus. Endoscopy also revealed features of mild chronic gastritis. Endoscopic diagnosis of papillomata was suggested; punch biopsy from two of the yellowish projections was performed and sent for histopathological examination.

### Histopathological examination

**Figure 1:**
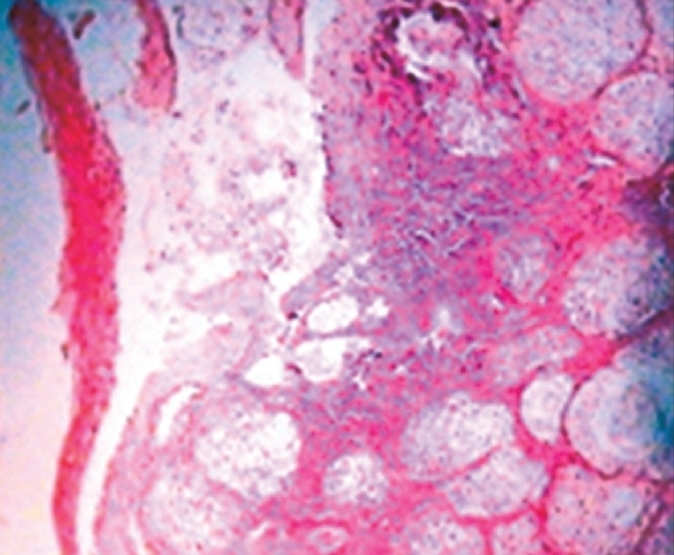
Microphotograph showing stratified squamous epithelium and sebaceous glands in lobules (H&E, ×100)

Microscopically, the non-keratinized stratified squamous epithelial lining showed minimal hyperplasia. The subepithelial area revealed lobulated sebaceous glands, deep in lamina propria. Each lobule consisted of polygonal cells with small nuclei and abundant clear cytoplasm [Figure 1]. No hair follicle was observed.

Mild lymphocytic infiltration was seen in the vicinity of the sebaceous glands. No histological features suggestive of reflux or infectious esophagitis were noted. Histological diagnosis of ectopic sebaceous glands in the esophagus was made.

The patient was treated symptomatically with antacids and omeprazole for one month, and he became symptom free and remained so at two years of follow up.

## DISCUSSION

The presence of ectopic sebaceous glands was first described in lips and oral cavity by Fordyce in1896 (the condition was called Fordyce's disease). Since then, it has been described in various other sites, such as eyes and orbits, palms and soles, parotid glands, and larynx.

However, in 1962, De La Pava and Pickren first reported the presence of sebaceous glands in an endodermally derived organ, the esophagus.[1] The authors reported such occurrences in 4 of the 200 unselected cadavers during an autopsy study.

Ectopic sebaceous glands in esophagus were first observed during endoscopy by Ramakrishnan and Brinker[5] in 1978 in a 44-year-old nonsmoker. The lesions may be single or multiple,[6] sometimes more than hundred.[2,3] Although no specific clinical symptoms attributable to esophageal sebaceous glands have been identified, some patients may experience symptoms such as heartburn and discomfort during swallowing.[2]

### Histogenesis

The most interesting aspect of this condition is histogenesis, which is not completely clear. Several hypotheses have been postulated.[4]

1. Metaplasia theory: This is favored by the occurrence of this condition in the elderly, and not in children, and because the arrangement of these glands are similar to that of esophageal submucosal glands.
2. Heterotopia theory (embryological misplacement): This theory is not supported by endoscopic studies and autopsy studies conducted on infants and children.[2]

There were no reports on any significant complications resulting from this condition. Endoscopic follow-up in such cases after a period of 8 months to 5 years showed no significant variation in the number or size of these lesions.[7] Moreover, malignant tumors arising from such ectopic sebaceous glands have never been reported.

Most of these lesions appear to be harmless, and only symptomatic treatment or anti-reflux treatment is advised.[2] Ectopic sebaceous glands should be differentiated from other submucosal tumors, and proliferative mucosal lesions such as granular cell tumor, leiomyoma and papilloma. In our case, microscopic features were very characteristic of sebaceous glands.

## CONCLUSION

Ectopic sebaceous glands in esophagus are a very rare occurrence. It probably represents a metaplastic change in the esophageal submucosal glands. Here, we reported this rare occurrence in a 65-year-old male.
